# Primary Adenocarcinoma of the Urethra: A Case Report and Review of the Literature

**DOI:** 10.1089/cren.2015.0026

**Published:** 2015-12-01

**Authors:** Abbas Basiri, Behzad Narouie, Mohammad Hossein Moghadasi, Mohammad Ghasemi-Rad, Reza Valipour

**Affiliations:** ^1^ Urology and Nephrology Research Center, Department of Urology and Renal Transplantation, Shahid Labbafinejad Medical Center, Shahid Beheshti University of Medical Sciences, Tehran, Iran.; ^2^ Department of Medical Laboratory, Shahid Labbafinejad Medical Center, Iranian Social Security Organization Tehran, Iran.; ^3^ Division of Interventional Radiology, Harvard Medical School, Massachusetts General Hospital, Boston, Massachusetts.

## Abstract

Primary adenocarcinoma of the urethra is rarely reported. We report a case of a 47-year-old male with symptoms of urinary obstruction started 2 years before diagnosis. Video-assisted urethrocystoscopy revealed a papillary mass almost obstructing the entire lumen with bleeding. Pathology report was consistent with primary adenocarcinoma of the urethra.

## Introduction

Primary urethral carcinoma (UC) is defined as a tumor that is first detected in the urethra unlike secondary UC that is a recurrence of tumor in the urethra after prior diagnosis and treatment of carcinoma somewhere else in the urinary tract. Most causes of secondary UCs are reported after radical cystectomy of the urinary bladder caused by bladder cancer.^1^ Primary UC is considered a rare cancer,^2–4^ accounting for <1% of all malignancies^3^ with an incidence rate of 4/1,000,000/year.^2^ The survival for superficial type is 83% and for deep type it is 36%.^4^ Most primary UCs are of transitional type and squamous cell type, with less than 5% being reported as adenocarcinoma.^5^ Here we report a patient with symptomatic urinary obstruction having a well-differentiated adenocarcinoma of the urethra.

## Case Report

**FIG. 1.**
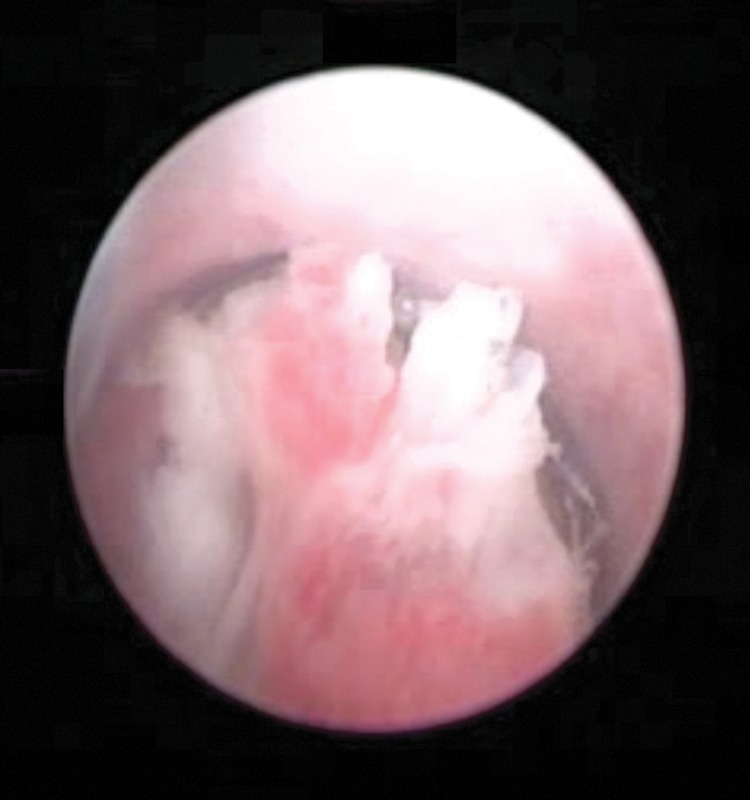
Video urethrocystoscopy showing papillary tumor at the level of bulbar urethra.

**FIG. 2.**
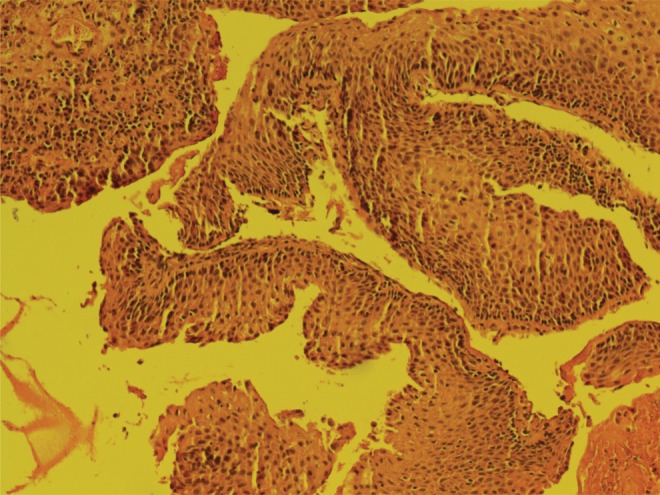
Papillary formed neoplastic tissue with atypical tall columnar epithelial, enlarged hyperchromatic nuclei, prominent nucleoli.

**FIG. 3.**
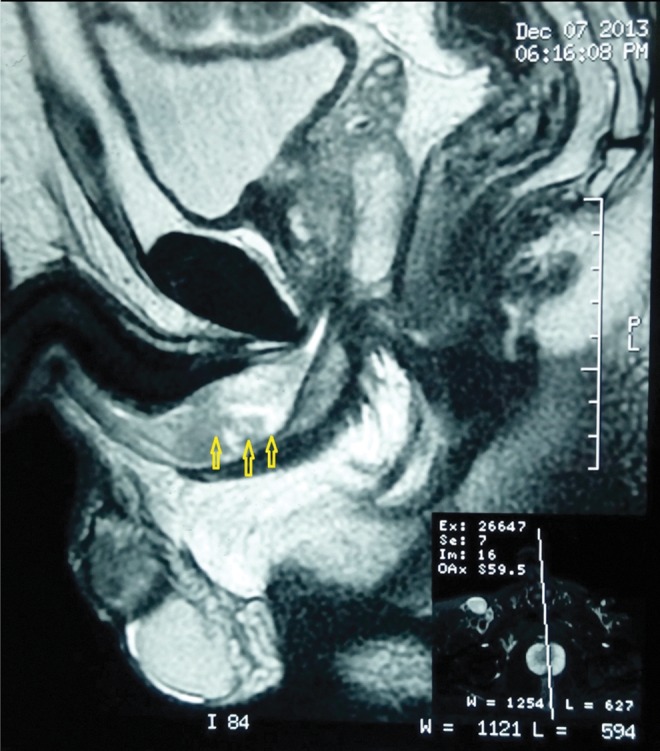
Sagittal T2 weighted shows a high signal large well-defined lesion at the bulbar portion of corpus spongiosum.

A 47-year-old male patient with 2-year history of symptomatic urinary obstruction was presented to our urology clinic at a tertiary referral hospital. He was also complaining of frequent gross terminal hematuria and lower urinary tract symptoms. Six months ago, he had total hematuria, and upon referring to a urologist, he was found to have urethral stricture and a retrograde urethrography, after which video urethrocystoscopy was performed (Fig. 1). Urethroscopy revealed mild penile urethral narrowing at the level of bulbar urethra, which was dilated. It showed a papillary tumor with bleeding, measuring 2 to 3 cm and almost filling the lumen. A sample was taken and sent to a pathologist. The membranous, prostatic urethra and bladder neck were intact as well as no pathology was seen inside the bladder. The pathology report showed papillary formed neoplastic tissue, in which atypical tall columnar epithelial with enlarged hyperchromatic nuclei and prominent nucleoli was clear. Cytoplasm and the underlying inflamed stroma infiltrated mostly by acute inflammatory cells, consistent with well-differentiated adenocarcinoma with focal mucin production (Fig. 2). The tumor had invaded into the submucosa and the deep margin is free of tumor. A pelvic MRI with and without contrast was performed to investigate possible metastasis (Fig. 3). A 44 × 19 mm intraurethral enhancing circumferential mass in the bulbar portion of the urethra was seen that causes expansion of urethra. The membranous part of the urethra was clear. There was no evidence of extracorporal invasion of the mass. Prostate gland with 55 cc volume was enlarged. The bladder had irregular border. The patient underwent surgery and the mass was removed. After 6 months of follow-up, the patient is fine and has no complaints.

## Discussion

Tumors of the male urethra are categorized according to location and histologic features.^6^ The UC is localized to the bulbomembranous urethra in 60% of the patients, penile urethra in 30% of patients, and prostatic urethra in 10% of patients.^7^ A recent population-based study, Surveillance, Epidemiology, and End Results (SEER), and analysis of 2065 men with primary UC (mean age: 73 years) showed that urothelial carcinoma (78%) was most common, and squamous cell carcinoma (SCC) (12%) and adenocarcinoma (5%) were significantly less frequent.^5^

For male primary UC, various predisposing factors have been reported, including urethral strictures,^2,4,8,9^ chronic irritation after intermittent catheterization or urethroplasty,^10–12^ external beam radiation therapy,^13^ radioactive seed implantation,^14^ chronic urethral inflammation urethritis after sexually transmitted diseases (i.e., condylomata associated with human papillomavirus 16),^4,15,16^ and congenital origin (i.e., clear cell adenocarcinoma).^17^

The histologic subtype of UC also varies by anatomic location. Carcinomas of the prostatic urethra are of transitional cell origin in 90% and of squamous cell origin in 10%; and carcinomas of the bulbomembranous urethra are of squamous cell origin in 80%, of transitional cell origin in 10%, and of adenocarcinoma or undifferentiated in 10%.^7^ Male UC can spread by direct extension to adjacent structures, usually involving the vascular spaces of the corpus spongiosum and the periurethral tissues, or it can metastasize through lymphatic embolization to regional lymph nodes. The lymphatic vessels from the anterior urethra drain into the superficial and deep inguinal lymph nodes and occasionally into the external iliac lymph nodes. Tumors of the posterior urethra most commonly spread to the pelvic lymph nodes. Palpable inguinal lymph nodes occur in about 20% of cases and almost always represent metastatic disease, in contrast to penile cancer, in which a large percentage of palpable nodes may be inflammatory. Hematogenous dissemination is uncommon except in advanced disease.^18^

When primary UC becomes clinically apparent, most patients (45%–57%) present with symptoms associated with locally advanced disease (T3/T4).^19,20^ At initial presentation, visible hematuria or bloody urethral discharge is reported in as much as 62% of the cases. Further symptoms of locally advanced disease include an extraurethral mass (52%), bladder outlet obstruction (48%), pelvic pain (33%), urethrocutaneous fistula (10%), abscess formation (5%), or dyspareunia.^20^

The onset of malignant change in a patient with chronic urethral stricture disease may be insidious, and a high index of clinical suspicion is necessary to diagnose these tumors expediently. The most common presenting symptoms are urethral bleeding, a palpable urethral mass, and obstructive voiding symptoms.^2^ In men, physical examination should comprise palpation of the external genitalia for suspicious indurations or masses and a digital rectal examination.^21^ Bilateral inguinal palpation should be conducted to assess the presence of enlarged lymph nodes, describing location, size, and mobility.^22^

The role of urinary cytology in primary UC is limited, and its sensitivity ranges between 55% and 59%.^23^ Detection rate depends on the underlying histologic entity. In male patients, the sensitivity for urothelial carcinoma and SCC was reported to be 80% and 50%, respectively.^21^

Depending on the depth of infiltration according to the TNM classification for UC, superficial tumors as papillary carcinoma *in situ* or at the basement membrane crossing are described as T1. The infiltration of the corpus spongiosum, prostate, or periurethral muscle is classified with T2. In addition, the involvement of the corpora cavernosa, the bladder neck, or a prostate capsule sunburst as T3 and an infestation of adjacent organs are defined as T4.^2^

The primary treatment is surgery. For UC in stage pT2, there are two curative surgical approaches: in the proximal urethra, this involves the prostatic urethra and membranous urethra. Should a penectomy with prostatectomy be necessary, it should be performed with cystoprostatectomy and removal of the pelvic lymph nodes.^2^

Earlier lesions of the bulbomembranous urethra have been treated effectively by transurethral resection or by segmental excision of the involved urethral segment with an end-to-end anastomosis. Unfortunately, cases appropriate for limited resection are rare.^7^

A statement on evidence-based therapies is difficult. The etiology may contribute to the development of a UC by several factors.^2^ In a distal localization, the bulbar, penile urethra, and navicular fossa comprising a penis-conserving surgery can be performed.^2^

Adenocarcinoma of the urethra, although rare, should be in deferential diagnosis of patients with a long history of urinary obstruction and gross hematuria.

## Abbreviations Used

MRI: magnetic resonance imaging
SCC: squamous cell carcinoma
UC: urethral carcinoma

## References

[1] Boorjian SA, Kim SP, Weight CJ, Cheville JC, Thapa P, Frank I Risk factors and outcomes of urethral recurrence following radical cystectomy. Eur Urol 2011;60:1266–1272 21871713 10.1016/j.eururo.2011.08.030

[2] Maek M [Penis-preserving surgery in patients with primary penile urethral cancer] (Ger). Urologe A 2014;53:1800–1804 25272986 10.1007/s00120-014-3583-4

[3] Gatta G, van der Zwan JM, Casali PG, et al. Rare cancers are not so rare: The rare cancer burden in Europe. Eur J Cancer 2011;47:2493–2511 22033323 10.1016/j.ejca.2011.08.008

[4] Dalbagni G, Zhang ZF, Lacombe L, Herr HW Male urethral carcinoma: Analysis of treatment outcome. Urology 1999;53:1126–1132 10367840 10.1016/s0090-4295(98)00659-1

[5] Rabbani F Prognostic factors in male urethral cancer. Cancer 2011;117:2426–2434 24048790 10.1002/cncr.25787

[6] Mostofi FK, Davis CJ Jr., Sesterhenn IA Carcinoma of the male and female urethra. Urol Clin North Am 1992;19:347–358 1574825

[7] Craig AP Campbel-Walsh Urology, 10th ed. Philadelphia: Elsevier Saunders, 2012

[8] Medina Perez M, Valero Puerta J, Sanchez Gonzalez M, Valpuesta Fernandez I, Marin Martin J [Squamous carcinoma of the male urethra, its presentation as a scrotal abscess] (Spa). Arch Esp Urol 1999;52:792–794 10540772

[9] Van de Voorde W, Meertens B, Baert L, Lauweryns J Urethral squamous cell carcinoma associated with urethral stricture and urethroplasty. Eur J Surg Oncol 1994;20:478–483 8076714

[10] Colapinto V, Evans DH Primary carcinoma of the male urethra developing after urethroplasty for stricture. J Urology 1977;118:581–584 10.1016/s0022-5347(17)58111-2 916053

[11] Mohanty NK, Jolly BB, Saxena S, Dawson L Squamous cell carcinoma of perineal urethrostomy. Urol Int 1995;55:118–119 8533195 10.1159/000282765

[12] Sawczuk I, Acosta R, Grant D, White RD Post urethroplasty squamous cell carcinoma. N Y State J Med 1986;86:261–263 3459083

[13] Mohan H, Bal A, Punia RP, Bawa AS Squamous cell carcinoma of the prostate. Int J Urol 2003;10:114–116 12588611 10.1046/j.1442-2042.2003.00580.x

[14] Arva NC, Das K Diagnostic dilemmas of squamous differentiation in prostate carcinoma case report and review of the literature. Diagn Pathol 2011;6:46 21627811 10.1186/1746-1596-6-46 PMC3118316

[15] Cupp MR, Malek RS, Goellner JR, Espy MJ, Smith TF Detection of human papillomavirus DNA in primary squamous cell carcinoma of the male urethra. Urology 1996;48:551–555 8886059 10.1016/S0090-4295(96)00246-4

[16] Wiener JS, Liu ET, Walther PJ Oncogenic human papillomavirus type 16 is associated with squamous cell cancer of the male urethra. Cancer Res 1992;52:5018–5023 1325290

[17] Gandhi JS, Khurana A, Tewari A, Mehta A Clear cell adenocarcinoma of the male urethral tract. Indian J Pathol Microbiol 2012;55:245–247 22771656 10.4103/0377-4929.97895

[18] Vapnek JM, Hricak H, Carroll PR Recent advances in imaging studies for staging of penile and urethral carcinoma. Urol Clin North Am 1992;19:257–266 1574816

[19] Derksen JW, Visser O, de la Riviere GB, Meuleman EJ, Heldeweg EA, Lagerveld BW Primary urethral carcinoma in females: An epidemiologic study on demographical factors, histological types, tumour stage and survival. World J Urol 2013;31:147–153 22614443 10.1007/s00345-012-0882-5

[20] Gheiler EL, Tefilli MV, Tiguert R, de Oliveira JG, Pontes JE, Wood DP Jr Management of primary urethral cancer. Urology 1998;52:487–493 9730466 10.1016/s0090-4295(98)00199-x

[21] Karnes RJ, Breau RH, Lightner DJ Surgery for urethral cancer. Urol Clin North Am 2010;37:445–457 20674699 10.1016/j.ucl.2010.04.011

[22] Blaivas JG, Flisser AJ, Bleustein CB, Panagopoulos G Periurethral masses: Etiology and diagnosis in a large series of women. Obstet and Gynecol 2004;103(5 Pt 1):842–847 10.1097/01.AOG.0000124848.63750.e6 15121554

[23] Touijer AK, Dalbagni G Role of voided urine cytology in diagnosing primary urethral carcinoma. Urology 2004;63:33–35 14751342 10.1016/j.urology.2003.08.007

